# Diagnostic performance of *T*_1_ and *T*_2_ mapping to detect intramyocardial hemorrhage in reperfused ST‐segment elevation myocardial infarction (STEMI) patients

**DOI:** 10.1002/jmri.25638

**Published:** 2017-02-15

**Authors:** Heerajnarain Bulluck, Stefania Rosmini, Amna Abdel‐Gadir, Anish N. Bhuva, Thomas A. Treibel, Marianna Fontana, Esther Gonzalez‐Lopez, Manish Ramlall, Ashraf Hamarneh, Alex Sirker, Anna S. Herrey, Charlotte Manisty, Derek M. Yellon, James C. Moon, Derek J. Hausenloy

**Affiliations:** ^1^ Hatter Cardiovascular Institute Institute of Cardiovascular Science University College London UK; ^2^ National Institute of Health Research University College London Hospitals Biomedical Research Centre UK; ^3^ Barts Heart Centre St Bartholomew's Hospital London UK; ^4^ Heart Failure and Inherited Cardiac Diseases Unit Department of Cardiology Hospital Universitario Puerta de Hierro Majadahonda, Manuel de Falla Madrid Spain; ^5^ Cardiovascular and Metabolic Disorders Program Duke‐National University of Singapore; ^6^ National Heart Research Institute Singapore, National Heart Centre Singapore

**Keywords:** ST‐segment elevation myocardial infarction, *T*_1_ mapping, *T*_2_ mapping, $T_{2}^{*}$ mapping, intramyocardial hemorrhage, microvascular obstruction

## Abstract

**Purpose:**

To investigate the performance of *T*_1_ and *T*_2_ mapping to detect intramyocardial hemorrhage (IMH) in ST‐segment elevation myocardial infarction (STEMI) patients treated by primary percutaneous coronary intervention (PPCI).

**Materials and Methods:**

Fifty STEMI patients were prospectively recruited between August 2013 and July 2014 following informed consent. Forty‐eight patients completed a 1.5T cardiac magnetic resonance imaging (MRI) with native *T*_1_, *T*_2_, and $T_{2}^{*}$ maps at 4 ± 2 days. Receiver operating characteristic (ROC) analyses were performed to assess the performance of *T*_1_ and *T*_2_ to detect IMH.

**Results:**

The mean age was 59 ± 13 years old and 88% (24/48) were male. In all, 39 patients had interpretable $T_{2}^{*}$ maps and 26/39 (67%) of the patients had IMH ( $T_{2}^{*}$ <20 msec on $T_{2}^{*}$ maps). Both *T*_1_ and *T*_2_ values of the hypointense core within the area‐at‐risk (AAR) performed equally well to detect IMH (*T*_1_ maps AUC 0.86 [95% confidence interval [CI] 0.72–0.99] versus *T*_2_ maps AUC 0.86 [95% CI 0.74–0.99]; *P* = 0.94). Using the binary assessment of presence or absence of a hypointense core on the maps, the diagnostic performance of *T*_1_ and *T*_2_ remained equally good (*T*_1_ AUC 0.87 [95% CI 0.73–1.00] versus *T*_2_ AUC 0.85 [95% CI 0.71–0.99]; *P* = 0.90) with good sensitivity and specificity (*T*_1_: 88% and 85% and *T*_2_: 85% and 85%, respectively).

**Conclusion:**

The presence of a hypointense core on the *T*_1_ and *T*_2_ maps can detect IMH equally well and with good sensitivity and specificity in reperfused STEMI patients and could be used as an alternative when $T_{2}^{*}$ images are not acquired or are not interpretable.

**Level of Evidence**: 2

**Technical Efficacy:** Stage 2

J. MAGN. RESON. IMAGING 2017;46:877–886

Primary percutaneous coronary intervention (PPCI) is the reperfusion strategy of choice in ST‐segment elevation myocardial infarction (STEMI). However, paradoxically, the process of reperfusion itself can lead to microvascular obstruction (MVO) and intramyocardial hemorrhage (IMH).^1^, ^2^ Nearly 50% of patients develop MVO,^3^ and 35–40% have IMH,^4^, ^5^ as detected by cardiac magnetic resonance (MR). Both MVO and IMH are associated with larger myocardial infarct (MI) sizes, adverse left ventricular (LV) remodeling and poor clinical outcomes, as recently summarized in two meta‐analyses.^3^, ^4^ Cardiac MR within the first week following STEMI using $T_{2}^{*}$ imaging has been shown to detect IMH in animal models of acute MI^6^, ^7^ and a threshold value of T* <20 msec has subsequently been used in several clinical studies as the reference for IMH.^2^, ^5^, ^8^, ^9^ Although $T_{2}^{*}$ imaging is currently the reference standard for the detection of IMH, it is prone to motion, flow and off‐resonance artifacts.^10^ In a recent study by Carrick et al,^5^ only 86% of patients had analyzable $T_{2}^{*}$ data. Furthermore they also showed that MVO and IMH are dynamic and follow distinct time courses and had different prognostic significance.^5^

*T*_2_‐weighted short tau inversion recovery (STIR) imaging, which has been used to delineate the area‐at‐risk (AAR) in reperfused STEMI patients, has also been used to detect IMH as the presence of a hypointense core within the AAR.^11^, ^12^, ^13^ However, STIR imaging has been shown to have a lower diagnostic performance for detecting IMH when compared to $T_{2}^{*}$ imaging.^7^, ^8^ Native *T*_1_ and *T*_2_ maps have been explored as alternative methods for quantifying the AAR in reperfused STEMI patients^14^, ^15^ and MVO and IMH can also manifest as a hypointense core on the native *T*_1_ 9, 16 and *T*_2_ maps.^7^, ^17^, ^18^ However, the diagnostic performance of native *T*_1_ and *T*_2_ maps to detect the presence of IMH and MVO following STEMI has not been directly compared. Therefore, the main aim of this study was to investigate the performance of hypointense core on the native *T*_1_ (referred to as *T*_1_ throughout the article for simplicity) and *T*_2_ maps to detect IMH within the first week in reperfused STEMI patients, using $T_{2}^{*}$ mapping as the reference standard for IMH.^2^, ^5^, ^6^, ^7^, ^8^, ^9^ Secondly, we also aimed to assess the performance of the hypointense core on the *T*_1_ and *T*_2_ maps to detect early and late MVO, using gadolinium enhancement as the reference standard for MVO.^12^, ^19^, ^20^, ^21^, ^22^

## Materials and Methods

### Study Population

The local Ethics Committee approved this study. The patients included in this study have been previously reported.^41^, ^42^, ^43^ In brief, between August 2013 and July 2014, consecutive patients were screened and 50 STEMI patients reperfused by PPCI within 12 hours of onset (diagnosis and treatment as per current guidelines^23^, ^24^ were prospectively recruited at one center. The main exclusion criteria were previous MI and standard recognized contraindications to cardiac MR such as estimated glomerular filtration rate <30 mL/min, ferromagnetic implants, and known claustrophobia.

### Cardiac MR Acquisition

Cardiac MR was performed on a 1.5T scanner (Magnetom Avanto, Siemens Medical Solutions, Erlangen, Germany) using a 32‐channel phased‐array cardiac coil. The imaging protocol included full left ventricular (LV) short axis coverage of cines, native *T*_1_ mapping, *T*_2_ mapping, three (basal, mid, and apical) LV short axis slices of $T_{2}^{*}$ mapping and full LV short axis coverage of early and late gadolinium enhancement (EGE and LGE). All the short axis images were aligned with the cine short axis slice position. Colored *T*_1_/T/ $T_{2}^{*}$ maps using the default look up table from the scanner were generated.

#### Native *T*_1_ mapping (Work In Progress 448B, Siemens Healthcare)

Native *T*_1_ maps were acquired with a steady‐state free precession (SSFP)‐based modified Look–Locker inversion recovery (MOLLI) sequence (flip angle = 35°; pixel bandwidth 977 Hz/pixel; matrix = 256 × 144; echo time = 1.1 msec; slice thickness 6 mm; uninterpolated resolution = 1.5 × 1.5 mm) using a 5s(3s)3s sampling protocol.^25^ Motion correction and a nonlinear least‐square curve fitting of the set of images acquired at different inversion times were performed inline by the scanner to generate a pixel‐wise colored *T*_1_ map.^26^

#### *T*_2_ mapping (Work In Progress 448B, Siemens Healthcare)

Colored *T*_2_ maps consisting of pixel‐wise *T*_2_ values were generated inline following motion correction and fitting to estimate *T*_2_ relaxation times^27^ after acquiring three single‐shot images (flip angle = 70°; pixel bandwidth 930 Hz/pixel; matrix = 116 × 192; echo time = 1.1 msec; repetition time = 3 × R‐R interval; slice thickness = 6 mm; uninterpolated resolution = 2.0 × 2.0 mm) at different *T*_2_ preparation times (0 msec, 24 msec, and 55 msec, respectively).

#### $T_{2}^{*}$ Mapping (Work In Progress 448B, Siemens Healthcare)

$T_{2}^{*}$ maps were obtained (bandwidth 814(×8) Hz/pixel; echo times × 8: 2.7, 5, 7.3, 9.6, 11.9, 14.2, 16.5, 18.8 msec; flip angle = 18°; matrix = 256 × 115; slice thickness = 8 mm; uninterpolated resolution = 1.6 × 1.6 mm) and a colored pixel‐wise $T_{2}^{*}$ map was generated inline by the scanner.

#### Early and Late Gadolinium Enhancement

EGE images were acquired 1–2 minutes after the injection of 0.1 mmol/kg of gadoterate meglumine (Gd‐DOTA marketed as Dotarem, Guerbet, Paris, France) using inversion recovery single‐shot SSFP *T*_1_‐weighted sequence at a fixed high inversion time of 440 msec^19^ (typical imaging parameters: bandwidth 898 Hz/pixel; echo time = 1.1 msec; repetition time = 700–900 msec; flip angle = 50°; acquisition matrix = 110 × 192; slice thickness = 8 mm; uninterpolated resolution = 2.1 × 2.1 mm). LGE images were acquired after 10–15 minutes, using either a standard segmented “fast low‐angle shot” 2D inversion‐recovery gradient echo sequence LGE phase sensitive inversion recovery (PSIR) sequence (in 25/48 patients; typical imaging parameters: bandwidth 140 Hz/pixel; echo time = 3.2 msec; repetition time = 700–900 msec; flip angle = 21°; acquisition matrix = 125 × 256; slice thickness = 8 mm; uninterpolated resolution = 1.6 × 1.6mm) or a respiratory motion‐corrected, free‐breathing single‐shot SSFP averaged PSIR sequence^28^ (in 23/48 patients; typical imaging parameters: bandwidth 977 Hz/pixel; echo time = 1.48 msec; repetition time = 700–900 msec; flip angle = 50°; acquisition matrix = 144 × 256; slice thickness = 8 mm: uninterpolated resolution = 1.6 × 1.6 mm).

### Cardiac MRI Analysis

Imaging analysis was performed using CVI42 software (v. 5.1.2[303], Calgary, Canada). The endocardial and epicardial borders were manually drawn and MI size was quantified in grams and as a percentage of the LV volume (%LV) using a signal intensity threshold of 5 standard deviations (SD)^29^ above the remote myocardium. Areas of hypointense core of MVO were manually included as part of the MI zone.

Although the *T*_1_ and *T*_2_ maps were performed with breath‐hold and motion‐correction, the raw images were visually assessed for any misalignment due to significant motion between single‐shot raw images, mistriggering, partial volume effects, and artifacts as previously described by von Knobelsdorff‐Brenkenhoff et al.^30^ The $T_{2}^{*}$ maps and their raw images were visually assessed for breathing, motion, and off‐resonance artifacts.

The endocardial and epicardial borders were manually drawn on the *T*_2_ maps and the AAR was obtained using a threshold of 2SD^31^, ^32^ above the remote myocardial *T*_2_ relaxation time and expressed as %LV. Areas of hypointense core within the areas of hyperenhancement were manually included as part of the MI zone.

**Figure 1:**
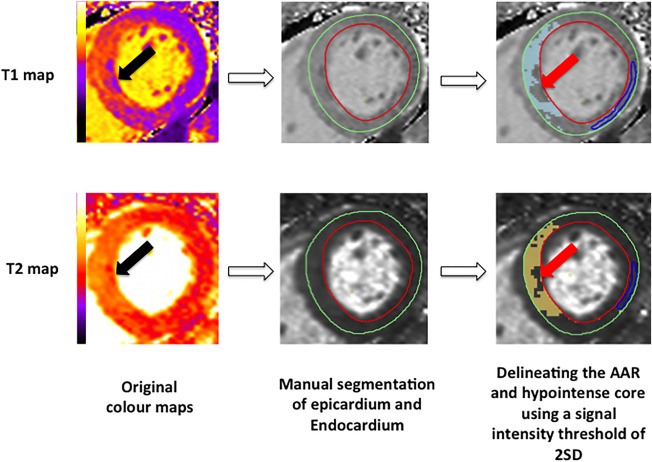
Semiautomated method used to identify the hypointense core on the *T*_1_ and *T*_2_ maps.

On the *T*_1_ and *T*_2_ maps matching the $T_{2}^{*}$ maps, the 2SD threshold was also used to identify the hypointense core (hypointense area within the hyperenhanced area with a subendocardial margin) on both maps to identify the AAR and the hypointense core (Fig. 1). So far, no studies have validated a cutoff threshold to detect IMH and MVO from these maps and we chose to use the same threshold used to quantify the AAR to delineate the hypointense core as a semiautomated method to minimize bias (Fig. 1). These areas were included as part of the MI zone and AAR. Regions of interest (ROIs) were manually drawn in the hypointense core (expressed as *T*_1Core_ and *T*_2Core_), the salvaged myocardium within the AAR (expressed as *T*_1Salvage_ and *T*_2Salvage_), and the remote myocardium (expressed as *T*_1Remote_ and *T*_2Remote_) on both maps. In cases when no hypointense core was identified, the ROI was drawn in the area of infarct. The *T*_1_ and *T*_2_ maps were also graded in a binary fashion to detect the presence or absence of a hypointense core (with the help of the semiautomated method as above).

Ten randomly selected patients were separately analyzed by two investigators, 2 months apart for intraobserver and interobserver variability.

Corresponding ROIs were drawn in the hypointense core of the $T_{2}^{*}$ maps and the remote myocardium (colocalized with the ROIs on the *T*_1_ and *T*_2_ maps) and representative values were recorded.

#### Cardiac MR Definitions

IMH was defined as a hypointense core on the $T_{2}^{*}$ maps with a $T_{2}^{*}$ <20 msec as previously validated^6^ and subsequently used in several clinical STEMI studies.^2^, ^5^, ^8^, ^9^

**Figure 2:**
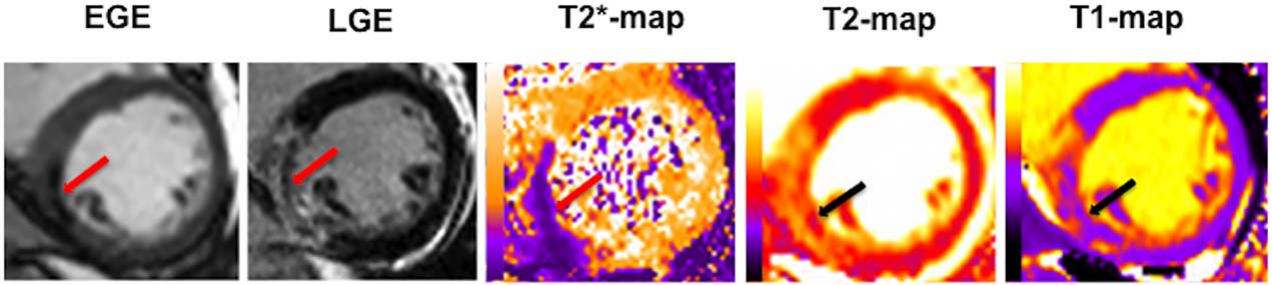
Example of a patient with an acute inferior MI depicting the evidence of MVO on EGE and LGE scans with corresponding hypointense cores (red and black arrows) on the basal LV short axis $T_{2}^{*}$, *T*_1_, and *T*_2_ maps.

Early MVO was defined as areas of dark core within the infarcted territory visually detected (red arrow on the EGE image in Fig. 2) on the EGE images as previously described.^12^, ^19^, ^20^

Late MVO was defined as areas of dark core within the areas of LGE (red arrow on the LGE image in Fig. 2) acquired 10–15 minutes postcontrast on the LGE images as previously described.^12^, ^21^, ^22^

The above definitions were used for the reference standard by cardiac MR for IMH, early MVO, and late MVO.

### Statistical Analysis

Statistical analysis was performed using SPSS v. 22 (IBM, Armonk, NY) and MedCalc for Windows v. 15.6.1 (Medcalc Software, Ostend, Belgium). Continuous data were expressed as mean ± SD or median (interquartile range) and compared using paired or unpaired tests where appropriate. Categorical data were reported as frequencies and percentages. Patients with no interpretable $T_{2}^{*}$ maps were excluded from the analysis. Receiver operating characteristic (ROC) analyses were used to assess the diagnostic performance for *T*_1_ and *T*_2_ maps for detecting IMH on the acute scans and were compared using the method described by Delong et al.^33^ Interobserver and intraobserver variability for *T*_1_ and *T*_2_ values of the hypointense core were assessed in 10 patients and expressed as an intraclass correlation coefficient (ICC) (95% confidence interval [CI]). Cohen's kappa was used to assess interobserver and intraobserver agreement for the binary assessment of the maps. All statistical tests were two‐tailed, and *P* < 0.05 was considered statistically significant.

## Results

**Figure 3:**
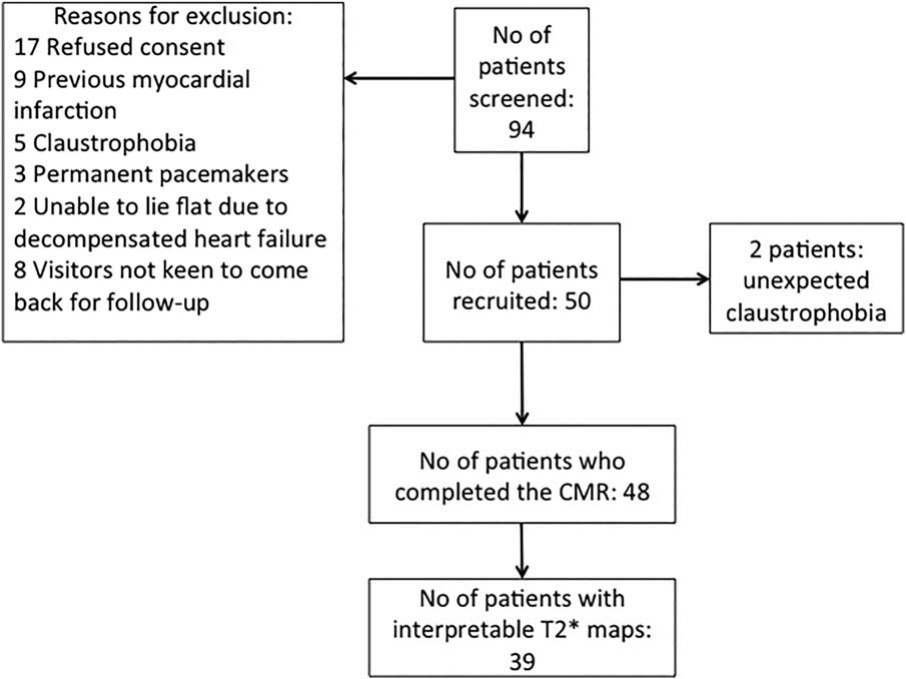
Details of the screening and recruitment of patients entering this study.

Figure 3 provides the details of the patients' screening and recruitment into the study. Forty‐eight patients with a mean age of 59 ± 13 years and 88% (42/48) male gender completed the cardiac MR study at 4 ± 2 days post‐PPCI. Patients' clinical characteristics are listed in Table 1. The median onset‐to‐balloon time was 182 (128–328) minutes. The mean MI size was 27.4 ± 14.6% of the LV and the AAR was 42.7 ± 11.9% of the LV. Early MVO was present in 79% (38/48) of patients and late in 63% (30/48) of patients.

**Table 1:** Clinical Characteristics of STEMI Patients

Details	Number
Number of patients	48
Male	40 (83%)
Age	58 ± 13
Diabetes mellitus	9 (19%)
Hypertension	15 (31%)
Smoking	15 (31%)
Dyslipidemia	15 (31%)
Chest pain onset to PPCI time (min)	182 (128‐328)
Infarct artery (%)	
LAD	28 (58%)
RCA	18 (38%)
Cx	2 (4%)
Pre‐PPCI TIMI flow (%)	
0	38(80%)
1	1 (2%)
2	4 (8%)
3	5 (10%)
Post‐PPCI TIMI flow (%)	
0	1 (2%)
1	0 (0%)
2	10 (21%)
3	37 (77%)

LAD: left anterior descending artery; RCA: right coronary artery; Cx: circumflex artery; TIMI: thrombolysis in myocardial infarction.

Ten percent of the *T*_1_ and *T*_2_ maps were not interpretable and were predominantly the basal or apical slices. All patients had a least one short axis *T*_1_ and *T*_2_ map matching the $T_{2}^{*}$ maps. $T_{2}^{*}$ maps were not interpretable in 19% (9/48) of the patients (due to motion flow and off‐resonance artifacts) but their corresponding *T*_1_ and *T*_2_ maps were interpretable.

The average scanning time was 59 ± 4 minutes, longer than a clinical cardiac MR scan, as on average an additional 15 minutes was required for *T*_1_ and *T*_2_ mapping acquisition (full LV coverage) and an additional 3 minutes for three short‐axis $T_{2}^{*}$ mapping acquisitions.

Figure 2 shows an example of the different imaging modalities acquired. These are mid‐LV short axis images of a patient with an acute inferior STEMI with MVO and the corresponding maps showing IMH (arrows).

A hypointense core was present on the *T*_2_ maps in 60% (29/48) of the patients and on the *T*_1_ maps in 63% (30/48) of the patients. Sixty‐seven percent (26/39) of the patients with interpretable $T_{2}^{*}$ maps had IMH.

### $T_{2}^{*}$ Mapping for the Detection of IMH (*n* = 39)

In patients with a hypointense core on the $T_{2}^{*}$ maps, the mean $T_{2}^{*}$ value of the core was 13 ± 3 msec compared to 33 ± 4 msec in the remote myocardium, *P* < 0.001. IMH occurred in 67%(26/39) of patients. As expected, patients with IMH were more likely to have larger MI size (33.4 ± 11.3% of the LV vs. 17.5 ± 9.8% of the LV, *P* < 0.001) and worse ejection fraction (47 ± 7% vs. 53 ± 7%, *P* = 0.04). Further details on the cardiac MR findings are summarized in Table 2.

**Table 2:** Cardiac MR Characteristics of STEMI Patients Divided Into Those With and Without IMH

	With IMH (*n* = 26)	Without IMH (*n* = 13)	*P* value
EDV/ml	172 ± 44	152 ± 17	0.06
ESV/ml	91 ± 30	73 ± 16	0.02^a^
EF/%	47 ± 7	53 ± 7	0.04^a^
LV Mass/g	117 ± 44	111 ± 23	0.66
Infarct size/ % of LV	33.4 ± 11.3	17.5 ± 9.8	<0.001^a^
Infarct size/ g	24.9 ± 8.6	11.4 ± 8.0	<0.001^a^
AAR/ %LV	46.5 ± 10.8	37.5 ± 13.3	0.03^a^
Late MVO/ % (*n*)	96 (25)	8 (1)	<0.001^a^
Early MVO/ % (*n*)	100 (26)	46 (6)	0.02^a^
T1_Core_/ ms	997 ± 79	1124 ± 65^b^	<0.001^a^
T1_Remote_/ ms	1035 ± 46	1014 ± 55^b^	0.03^a^
T1_Salvage_/ ms	1244 ± 79	1267 ± 65	0.43
T2_Core_/ ms	50 ± 4	57 ± 4	<0.001^a^
T2_Remote_/ ms	51 ± 3	50 ± 3	0.35
T2_Salvage_/ ms	66 ± 6	66 ± 7	0.85

a Denotes statistically significant.

b Includes patients with and without a detectable hypointense core.

IMH: intramyocardial hemorrhage; EDV: end diastolic volume; ESV: end systolic volume; EF: ejection fraction; LV: left ventricular mass; AAR: area‐at‐risk; MVO: microvascular obstruction.

### Detection of IMH by *T*_1_ and *T*_2_ mapping (*n* = 39)

**Figure 4:**
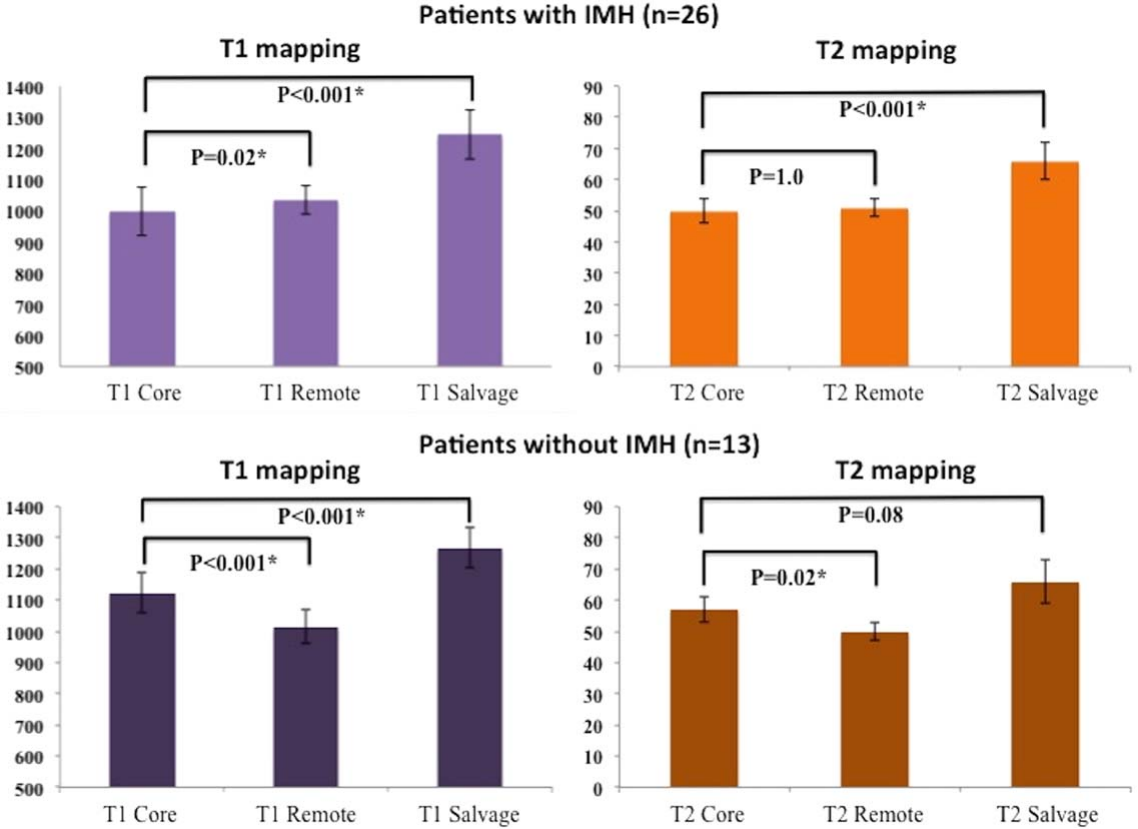
*T*_1_ and *T*_2_ values of the hypointense core, remote myocardium, and the salvaged myocardium. *Statistically significant difference.

**Figure 5:**
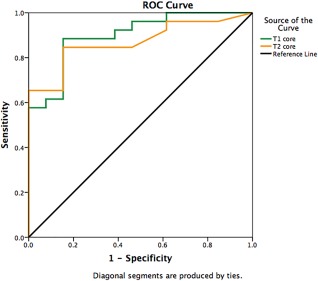
ROC curves for the diagnostic performance of *T*_1_ and *T*_2_ mapping to detect IMH on the acute scans when compared to $T_{2}^{*}$ maps

In patients with IMH, *T*_1Core_ was lower than *T*_1Remote_ (997 ± 79 msec vs. 1035 ± 46 msec, *P* = 0.02) whereas *T*_2Core_ was similar to *T*_2Remote_ (50 ± 4 msec vs. 51 ± 3 msec, *P* = 1.0). In patients without IMH, *T*_1Core_ and *T*_2Core_ were higher than *T*_1Remote_ and *T*_2Remote_ as shown in Fig. 4. ROC analyses of the *T*_1_ and *T*_2_ values of the hypointense core showed that both mapping techniques performed equally well at detecting IMH on the acute scans (*T*_1_ maps: area under the curve (AUC) 0.86 [95% CI 0.72‐0.99], cutoff value for *T*_1Core_: <1080 msec; *T*_2_ maps: AUC 0.86 [95% CI 0.74‐0.99]; *P* = 0.94, cutoff value for *T*_2Core_: <54 msec) (Fig. 5). When using the binary assessment of either presence or absence of a hypointense core on the *T*_1_ and *T*_2_ maps as a measure to detect IMH, *T*_1_ and *T*_2_ performed as well as the quantitative assessment of the maps (*T*_1_: AUC 0.87 [95% CI 0.73‐1.00], *T*_2_: AUC 0.85 [95% CI 0.71‐0.99]; *P* = 0.90). The presence of a hypointense core had a sensitivity of 88% and a specificity of 85% to detect IMH on the *T*_1_ maps and a sensitivity of 85% and specificity of 85% on the *T*_2_ maps. The accuracy by *T*_1_ mapping was 87% and 85% by *T*_2_ mapping. The positive predictive value by *T*_1_ and *T*_2_ mapping were both 92%. The negative predictive value was highest by *T*_1_ mapping at 87% and 85% by *T*_2_ mapping.

### Detection of Early and Late MVO by *T*_1_ and *T*_2_ Maps (*n* = 48)

The AUC for the presence of a hypointense core on the maps to detect early MVO was 0.83 (95% CI 0.70–0.97) for *T*_1_ (sensitivity: 76%; specificity: 90%) and 0.82 (95% CI 0.69–0.97) for *T*_2_ (sensitivity: 77%; specificity: 90%). For the detection of late MVO the AUC was 0.79 (95% CI 0.64–0.95) for *T*_1_ (sensitivity: 84%; specificity: 71%) 0.80 (95% CI 0.62–0.93) for *T*_2_ (sensitivity: 80%; specificity of 72%).

Table 3 summarizes further details on the diagnostic performances of *T*_1_ and *T*_2_ maps to detect IMH, early MVO, and late MVO.

**Table 3:** Summary of the Diagnostic Performances of T1 and T2 Maps to Detect IMH, Early MVO, and Late MVO

	Sensitivity/ %	Specificity/ %	Positive predictive value/ %	Negative predictive value/ %	Accuracy/ %
IMH					
T1 map	88	85	92	79	87
T2 map	85	85	92	73	85
Early MVO					
T1 map	76	90	97	50	79
T2 map	74	90	97	47	77
Late MVO					
T1 map	84	71	87	72	81
T2 map	80	72	86	68	79

IMH: intramyocardial hemorrhage; MVO: microvascular obstruction.

### ROC Comparison for the Detection of IMH, early MVO, and Late MVO

There was no significant difference in the diagnostic performance for *T*_1_ and *T*_2_ mapping to detect IMH, early MVO, and late MVO (*P* values for ROC curves comparison for IMH vs. early MVO, IMH vs. late MVO, early MVO vs. late MVO for *T*_1_ mapping: 0.90, 0.43, 0.37, respectively; for *T*_2_ mapping: 0.81, 0.58, and 0.42 respectively)

### Early MVO With and Without IMH (*n* = 32)

Thirty‐two patients had analyzable $T_{2}^{*}$ maps and early MVO. Eighty‐one percent (26/32) of the patients had early MVO with IMH and 19% (6/32) of the patients had early MVO without IMH. Both *T*_1_ and *T*_2_ values were significantly lower in patients with early MVO with IMH compared to those with early MVO without IMH (*T*_1Core_: 998[954–1036] msec vs. 1116[1085–1168], *P* < 0001; *T*_2Core_: 50[48–53] msec vs. 55[54–56] msec, *P* = 0.005). The same comparison was not performed for late MVO as only one patient had late MVO without IMH. All patients with early MVO with IMH developed late MVO compared to 2/6 (33%) of those with early MVO without IMH had late MVO, *P* < 0.0001.

### Interobserver and Intraobserver Variability (*n* = 10)

On the quantitative mapping analysis of the hypointense core, for intraobserver variability the ICC for the quantification of the hypointense core for the *T*_1_ maps was 0.944 (0.792–0.986) and for *T*_2_ maps was 0.903 (0.637–0.976). For interobserver variability, the ICC for *T*_1_ maps was 0.935 (0.746–0.984) and for *T*_2_ maps was 0.887 (0.528–0.972). On the qualitative mapping analysis for the hypointense core detection using the semiautomatic method, the interobserver and intraobserver agreement was 100%, Cohen's kappa = 1.0, *P* < 0.0001.

## Discussion

We have shown that the presence of a hypointense core within the area if hyperenhancement (AAR) on the *T*_1_ and *T*_2_ maps obtained at day 4 following a reperfused STEMI detected the presence of IMH with good specificity and sensitivity compared to the reference standard method using $T_{2}^{*}$ maps. The binary assessment of presence or absence of a hypointense core performed as well as the quantitative assessment of the actual *T*_1_ and *T*_2_ values of the hypointense core. However, $T_{2}^{*}$ maps are still the modality of choice when available, as the accuracy for *T*_1_ and *T*_2_ maps to detect IMH was only 85 to 87%, respectively. *T*_1_ and *T*_2_ maps provide an alternative option for the detection of IMH when $T_{2}^{*}$ imaging is not available.

The presence of a hypointense core on the *T*_1_ and *T*_2_ mapping performed equally well to detect early and late MVO. This is not surprising for late MVO, as most patients with late MVO also had IMH and therefore this approach performs well to detect hemorrhagic MVO in our cohort. Although numerically the AUCs were higher for the detection of early MVO than late MVO, ROC curves comparison did not show a statistical difference. *T*_1_ and *T*_2_ mapping could differentiate between early MVO with and without IMH. Furthermore, those with IMH were more likely to display late MVO. Early MVO likely represents a spectrum of etiologies for microvascular injury and late MVO represents the more severe form as IMH. A recent study^34^ using a porcine model of MI showed that *T*_2_‐STIR imaging could not discriminate between IMH and MVO but imaging was performed at 8 days and no $T_{2}^{*}$ data were acquired. Given the recent evidence of the dynamic nature for the detection of the paramagnetic properties of IMH,^35^ it is not possible to put the results of that study into context with our findings.

*T*_1_ of the infarct core was recently shown to be more prognostic than LV ejection fraction, infarct core *T*_2_, and IMH in a large cohort of STEMI patients.^9^ In a separate article of the same cohort of patients, Carrick et al^5^ showed that IMH was more closely associated with adverse LV remodeling than late MVO, but their timing of cardiac MR was a mean of 2.1 days and 87% of their patients with MVO had IMH. They also demonstrated the dynamic nature of MVO and IMH peaked at 2.9 days.^5^ So far, in other studies using $T_{2}^{*}$ for detection of IMH, CMR were performed at 2–3 days (O'Regan et al^2^: Day 3; Mather et al^19^: Day 2; Kali et al^7^: Day 3: Kandler et al^8^: Day 3; Zia et al^17^: Day 2). Whether performing cardiac MR ≥3 days post‐PPCI (our study: mean of 4 days—96% with MVO had IMH) may reveal more patients with MVO and IMH, which would have more prognostic significance, remains to be tested in future adequately powered studies.

The hypointense core on the *T*_2_ maps in reperfused STEMI patients has been noted in previous studies and was thought to be due to IMH.^17^, ^32^ Pedersen et al^36^ previously showed that *T*_1_‐weighted inversion recovery images could detect IMH with high sensitivity and specificity in a porcine model of acute STEMI. However, we are the first study to directly compare the diagnostic performance of *T*_1_ and *T*_2_ mapping to detect IMH against $T_{2}^{*}$ mapping in the clinical setting.

The mechanism of the low signal within the areas of IMH has previously been attributed to the paramagnetic properties of hemoglobin breakdown products.^20^ However, degradation of the extravasated erythrocytes to oxyhemoglobin, deoxyhemoglobin, and methemoglobin (strongly paramagnetic) is dynamic and would exhibit different *T*_1_ and *T*_2_ properties at various stages as previously described by Bradley et al^37^ in brain imaging. *T*_2_ was better at identifying deoxyhemoglobin, whereas *T*_1_ was better at detecting methemoglobin^37^ and this may explain the difference in sensitivities for *T*_1_ and *T*_2_ maps to detect IMH in our study. Breakdown of the erythrocyte membrane eventually leads to ferritin and hemosiderin deposits within macrophages.^20^ Although $T_{2}^{*}$ is the most sensitive to detect IMH, it is prone to motion artifacts due to relatively long breath‐hold duration and this has led to the development of free‐breathing $T_{2}^{*}$ mapping using motion corrected averaging.^10^ However, this is not widely available yet and therefore *T*_1_ and *T*_2_ mapping, which is increasingly becoming available in most centers performing STEMI research, may be an alternative option to assess for IMH and MVO. This approach would minimize patients dropping out of studies when $T_{2}^{*}$ mapping was not acquired or were not interpretable.

The mechanism for a hypointense core on the *T*_1_ and *T*_2_ maps in patients without IMH but with MVO is not clear. It has been postulated that this may be due to a localized reduction in tissue water content due to obstruction of the capillaries by distal embolization and plaques and cellular debris and compression from extrinsic edema.^5^, ^32^ The alternative explanation could be that the hypointense core on the *T*_1_ and *T*_2_ maps may still represent IMH but the hemoglobin degradation products are not paramagnetic enough to be detected by $T_{2}^{*}$ if imaged too early; more work remains to be done.

### Limitations

This was a small study of 39 patients and no formal power calculation was performed, but was similar in size to previous studies^7^, ^19^, ^38^, ^39^ and the large prevalence of IMH in our cohort may have been due to chance. Using a more conservative prevalence for IMH of 40% (expected AUC 0.85), the sample size required in prospective studies would need to be 85 patients (PASS 14 Power Analysis and Sample Size Software; 2015; NCSS, Kaysville, Utah) and almost double the number if the performance of the hypointense core on the *T*_1_ and *T*_2_ maps were assessed to differentiate between MVO with and without IMH. A large number of the $T_{2}^{*}$ maps were not interpretable in our study predominantly due to motion, flow, and off‐resonance artifacts and this highlights the challenge of performing a comprehensive cardiac MR scan with multiparametric mapping in acutely unwell STEMI patients (average scanning time of 1 hour) and also shown in a recent large study with 14% of $T_{2}^{*}$ maps being not analyzable.^5^ We did not quantify the extent of IMH, as whole coverage for $T_{2}^{*}$ were not available and this was not the aim of this study, and some patients with small areas of IMH may have been missed. Histological validation for the low *T*_1_ and *T*_2_ of the hypointense core was not possible in this study and warrants further investigation. We used two LGE readouts to accommodate for patients who preferred not to breath‐hold for the LGE acquisition given the long duration of the scan, and the difference in signal‐to‐noise ratio between the two may have affected the detection of MVO. The large majority of our patients with MVO also had IMH. Therefore, we could not assess whether the hypointense core on the *T*_1_ and *T*_2_ maps could differentiate between late MVO with IMH and late MVO without IMH. However, we did find a difference in the *T*_1Core_ and *T*_2Core_ between those with early MVO with IMH and early MVO without IMH and this needs to be confirmed in future studies. We did not acquire data on black blood *T*_2_‐STIR images or susceptibility‐weighted cardiac MRI (which has been shown to improve the detection of IMH at 1.5 T^40^ and 3T^38^ for comparison. The reduction in *T*_1_ and *T*_2_ using SSFP readouts could have been due to a combination of both the on‐resonance and off‐resonance signals from the paramagnetic components of the IMH and this was not elucidated in this study.

In conclusion, the presence of a hypointense core on *T*_1_ and *T*_2_ maps within the first week following a STEMI can detect IMH equally well and with good sensitivity and specificity. The *T*_1_ and *T*_2_ mapping techniques provide an alternative approach for the detection of IMH in situations when $T_{2}^{*}$ maps are not interpretable or not available. However, $T_{2}^{*}$ mapping currently remains the reference standard in the clinical setting and *T*_1_ and *T*_2_ mapping therefore may play a complementary role in future studies targeting IMH.
